# Curcumin Suppresses Hepatic Stellate Cell-Induced Hepatocarcinoma Angiogenesis and Invasion through Downregulating CTGF

**DOI:** 10.1155/2019/8148510

**Published:** 2019-01-16

**Authors:** Shan Shao, Wanxing Duan, Qinhong Xu, Xuqi Li, Liang Han, Wei Li, Dong Zhang, Zheng Wang, Jianjun Lei

**Affiliations:** ^1^From the Department of Oncology, First Affiliated Hospital of Xi'an Jiaotong University, 277 West Yanta Road, Xi'an, 710061 Shaanxi Province, China; ^2^From the Department of Hepatobiliary Surgery, First Affiliated Hospital of Xi'an Jiaotong University, 277 West Yanta Road, Xi'an, 710061 Shaanxi Province, China; ^3^From the Department of General Surgery, First Affiliated Hospital of Xi'an Jiaotong University, 277 West Yanta Road, Xi'an, 710061 Shaanxi Province, China

## Abstract

Microenvironment plays a vital role in tumor progression; we focused on elucidating the role of hepatic stellate cells (HSCs) in hepatocarcinoma (HCC) aggressiveness and investigated the potential protective effect of curcumin on HSC-driven hepatocarcinoma angiogenesis and invasion. Our data suggest that HSCs increase HCC reactive oxygen species (ROS) production to upregulate hypoxia-inducible factor-1*α* (HIF-1*α*) expression to promote angiogenesis, epithelial to mesenchymal transition (EMT) process and invasion. And HSCs could secrete soluble factors, such as interleukin-6 (IL-6), vascular endothelial cell growth factor (VEGF), and stromal-derived factor-1 (SDF-1) to facilitate HCC progression. Curcumin could significantly suppress the above HSC-induced effects in HCC and could abrogate ROS and HIF-1*α* expression in HCC. HIF-1*α* or connective tissue growth factor (CTGF) knockdown could abolish the aforementioned curcumin affection. Moreover, CTGF is a downstream gene of HIF-1*α*. In addition, nuclear factor E2-related factor 2 (Nrf2) and glutathione (GSH) are involved in curcumin protection of HCC. These data indicate that curcumin may induce ROS scavenging by upregulating Nrf2 and GSH, thus inhibiting HIF-1*α* stabilization to suppress CTGF expression to exhibit its protection on HCC. Curcumin has a promising therapeutic effect on HCC. CTGF is responsible for curcumin-induced protection in HCC.

## 1. Introduction

Hepatocellular carcinoma (HCC) represents the fifth most common cancer worldwide and is the third most common cause of cancer-related deaths [1]. Its high tendency to metastasize is considered to partially account for the extremely poor clinical prognosis of HCC. HCCs are typically highly vascularized [2, 3]. Transarterial chemoembolization (TACE) using the anthracycline antibiotic doxorubicin is the standard treatment for unresectable intermediate HCC and has survival benefit in asymptomatic patients with multifocal disease without vascular invasion or extrahepatic spread. Sorafenib, lenvatinib, which is noninferior to sorafenib, and regorafenib increase survival and are the standard treatments in advanced hepatocellular carcinoma. However, several clinical trials have revealed that sorafenib has limited anticancer effects to improving patient survival [4, 5]. Thus, it is an urgent need for a greater understanding of the molecular mechanism of HCC progression and seeking for new therapeutic targets for the treatment.

The stroma is closely involved in both hepatic fibrosis and carcinogenesis and is a vital player in the cellular and molecular mechanisms associated with these processes [6, 7]. Hepatic stellate cells (HSCs) are an important component in the liver, and its activation with subsequent phenotypic alterations is a critical event for fibrosis. Besides, HSCs can affect the initiation and progression of HCC. Previous studies have revealed that HSCs facilitate cancer cell invasion and proliferation through secreting growth factors and cytokines [8]. In addition, HSCs exhibit biological effect on regulating immune evasion and angiogenesis.

Curcumin, commonly known as turmeric, is a polyphenol derived from the *Curcuma longa* plant. It has been broadly used for centuries [9, 10], on account of its nontoxic and various therapeutic properties including antiseptic activity, antioxidant, and anti-inflammatory [9]. Recent studies have shown that curcumin exhibits anticancer activities through its effect on some biological pathways associated with cell cycle regulation, tumorigenesis, and metastasis [11, 12]. Curcumin has an inhibition effect on the transcription factor nuclear factor-*κ*B (NF-*κ*B) [13] and its downstream gene products (including cyclooxygenase-2, matrix metalloproteinase (MMP-9), interleukins, and Cyclin D1) [14, 15]. Moreover, curcumin regulates the expression of many growth cell adhesion molecules and factor receptors linking tumor angiogenesis and metastasis [9, 16].

Here, we investigated whether oxidative stress plays a vital role in HCC progression and evaluated the potential beneficial effects of curcumin on HSC-induced HCC invasiveness and angiogenesis. We revealed that curcumin has a promising therapeutic effect on HSC-induced HCC invasion and angiogenesis. CTGF is responsible for curcumin induce protection in HCC. Curcumin may induce ROS scavenging by upregulating Nrf2 and GSH, thus inhibiting HIF-1*α* stabilization to suppress CTGF expression to exhibit its protection on HCC.

## 2. Materials and Methods

### 2.1. Cell Lines and Cell Culture

The HCC cell line (HepG2) and human umbilical vein endothelial cells (HUVECs) were obtained from the Shanghai Institution for Biological Science (Shanghai, China). Human hepatic stellate cell lines (HSCs) were purchased from ScienCell Research Laborotary (Carlsbad, CA, USA). All cell lines were cultured at 37°C, 5% CO2, and 95% air in Dulbecco's modified Eagle's medium (DMEM) (high glucose) (HyClone, Logan, USA) containing 10% heat-inactivated fetal bovine serum (FBS) plus 100 *μ*g/ml ampicillin and 100 *μ*g/ml streptomycin.

### 2.2. Reagents

Anti-HIF-1*α* was obtained from Bioworld (St. Louis, MO, USA). The other antibodies, namely, anti-E-cadherin, anti-MMP-9, anti-vimentin, anti-CTGF, anti-Nrf2, and anti-*β*-actin, were purchased from Cell Signaling Technology Biotechnology (Danvers, MA, USA). Curcumin was purchased from Sigma-Aldrich (St. Louis, MO, USA) and DCF-DA was purchased from Molecular Probes (Eugene, OR, USA).

### 2.3. Real-Time Quantitative PCR (RT-qPCR)

TRIzol reagent was used to isolate total RNA, and the reverse transcription was developed using a PrimeScript RT reagent Kit (TaKaRa, Dalian, China). The iQ5 Multicolor Real-Time PCR Detection System (Bio-Rad, Hercules, CA, USA) was carried out to perform real-time PCR. The △△CT method was applied to determine fold changes in gene expression as normalized to those of GAPDH. The following PCR primers were as follows: MMP-9, forward: 5′-GAACCAATCTCACCGACAGG-3′ and reverse: 5′-GCCACCCGAGTGTAACCATA-3′; E-cadherin, forward: 5′-ATTCTGATTCTGCTGCTCTTG-3′ and reverse: 5′-AGTCCTG GTCCTCTTCTCC-3′; vimentin, forward: 5′-AATGACCGCTTCGCCAAC-3′and reverse: 5′-CCGCATCTCCTCCTCGTAG-3′; VEGF, forward: 5′-TGCAGATTATGCGGATCAAACC-3′ and reverse: 5′-TGCATTCACATTT GTTGTGCTGTAG-3′; HIF-1*α*, forward: 5′-AAGTCTAGGGATGCAGCA-3′ and reverse: 5′-CAAGATCACC AGCATCATG-3′; IL-6, forward: 5′-AGTTCCTGCAGTCCAGCCTGAG-3′ and reverse: 5′-TCAAACTGCATAGCCACTTTC C-3′; CTGF, forward: 5′-ACCTGTGGGATGGGCATCT-3′ and reverse 5′-CAGGCGGCTCTGCTTCTCTA-3′; and GAPDH, forward: 5′-ACCACAGTCCATGCCATCAC-3′ and reverse: 5′-TCCACCACCCTGTTGCTGAT-3′.

### 2.4. Western Blot Analysis

Total cellular protein of indicated cells was extracted and heated for 15 min at 75°C. 100 *μ*g of cellular proteins was separated on a 10% SDS-PAGE gel and then transferred to the PVDF membranes. The membranes were incubated with the following primary antibodies: anti-HIF-1*α*, anti-E-cadherin, anti-MMP-9, and anti-vimentin. The membrane was subsequently incubated with HRP-conjugated secondary antibodies, and an enhanced chemiluminescence detection system was used to perform the peroxidase reaction to visualize the immunoreactive bands.

### 2.5. HUVEC Tubule Formation Assay

200 *μ*l of Matrigel was used to coat each well of a 24-well plate. HUVECs (2 × 10^4^) from each group were resuspended into 200 *μ*l of conditioned media (CM) in each well and incubated at 37°C under 5% CO_2_ for 24 h. A light microscope were used to capture the image under at 100x magnification, and the number of capillary tubes were measured by calculating the total tube length of each image. We randomly chose three different fields per well and photographed the image using a light microscope. The total length of all tubing with each field was measured after calibration with a stage micrometer, and Prism 5 software (GraphPad Software, San Diego, CA, USA) was used to analyze the data.

### 2.6. Cell Invasion Assay

A chamber-based invasion assay (Millipore Co., Billerica, MA, USA) was used to determine HCC cell invasion. The upper surface of the membrane was coated with 25 ml of Matrigel (BD Biosciences, Franklin Lakes, NJ, USA). HepG2 cells (1 × 10^5^) from the indicated groups were resuspended in the upper chamber in serum-free media to allow migration towards a serum gradient (10%) in the lower chamber for 20 h. The noninvading cells were scraped from the top of the Matrigel, and the invading cells on the bottom surface were fixed with 4% paraformaldehyde and stained with crystal violet. The numbers of migrated cells were calculated in ten randomly selected fields under a light microscope at ×100 magnification.

### 2.7. Assay of Intracellular ROS

Intracellular H_2_O_2_ production assay is described in previous publications [17, 18]. Briefly, 5 *μ*g/ml DCF-DA was used to incubate the cells from the indicated groups for 5 minutes and then 1 ml of RIPA buffer was used to lyse the cells after washing with PBS. Fluorimetric analysis at 510 nm was applied to detect H_2_O_2_ concentrations. The data were normalized to total protein content.

### 2.8. RNA Interference

A negative control shRNA (sc-108060) (Santa Cruz, Dallas, Texas, USA) and shRNA against HIF-1*α* (sc-400036) (Santa Cruz) were obtained from Santa Cruz Biotechnology and were applied to transfect the HCC cells. RNA interference was performed using Lipofectamine (Invitrogen, Carlsbad, CA, USA), according to the manufacturer's instructions. After interference, puromycin was used to select the silenced cells. Then, the stably transfected cells were selected for further use.

### 2.9. Enzyme-Linked Immunosorbent Assay (ELISA)

HCC cells from the indicated groups were incubated with serum-free medium for 72 h. The concentrations of IL-6, VEGF, and SDF-1 in the CM were detected using an enzyme-linked immunosorbent assay (ELISA) kit (R&D, Minneapolis, MN, USA), according to the manufacturer's instructions.

### 2.10. Measurement of Glutathione Content

GSH and GSSG levels were measured in CGN extracts using the GSH reductase enzyme method. This assay is based on the reaction of GSH and thiol-mediated which produces the 5,5′-dithio-bis (2 nitrobenzoic acid) (DTNB) to 5-thio-2-nitrobenzoic acid (TNB), detectable at *λ* = 412 nm. The test is specific to GSH due to the specificity of the GSH reductase enzyme to GSH: the rate of accumulation of TNB is proportional to the concentration of GSH in the sample. Briefly, cell extract was diluted 1 : 2 with KPE buffer (0.1 M potassium phosphate, 5 mM disodium EDTA, pH 7.5) prior to the addition of freshly prepared DTNB (2.5 mM) and GSH reductase solutions (250 U/ml). Following the addition of *β*-NADPH, the absorbance (*λ* = 412 nm) was measured immediately at 30 s intervals for 2 min. The rate of change in absorbance was compared to that for GSH standards. The measurement of GSSG in each sample was identical to that used for the measurement of GSH, but with a previous treatment of the sample with 2-VP, which reacts out with GSH.

### 2.11. Statistical Analysis

The data were presented as the mean ± SD from at least three independent experiments. Statistical comparisons of more than 2 groups were performed using one-way analysis of variance with Bonferroni's post hoc test. Statistical comparisons between 2 samples were performed using the Student's *t*-test. Significance was defined as *p* < 0.05.

## 3. Results

### 3.1. Curcumin Suppresses HCC Angiogenesis Induced by HSCs through HIF-1*α*

**Figure 1:**
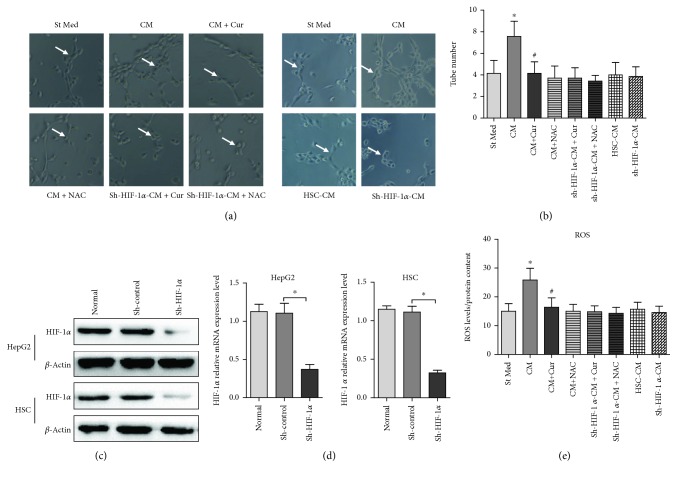
Curcumin inhibits HSC-induced HCC angiogenesis by suppressing HIF-1*α*. HUVECs were incubated with conditioned medium from the HepG2 (St Med), HepG2 + HSC (CM), HepG2 + HSC + curcumin (CM + Cur), HepG2 + HSC + NAC (CM + NAC), sh-HIF-1*α*-HepG2 + HSC + curcumin (sh-HIF-1*α*-CM + Cur), sh-HIF-1*α*-HepG2 + HSC + NAC (sh-HIF-1*α*-CM + NAC) groups, HSC only (HSC-CM), and sh-HIF-1*α*-HepG2 only (sh-HIF-1*α*-CM). Cur stands for Curcumin. 50 *μ*M Curcumin was added into the medium for 24 h in CM + Cur group or sh-HIF-1*α*-CM + Cur group. 20 mM NAC was added into the medium for 24 h in CM + NAC group or sh-HIF-1*α*-CM + NAC group. (a) Angiogenesis was evaluated based on tube formation (indicated by arrows). (b) Tube numbers were counted. ^∗^*p* < 0.05 versus St Med group (*n* = 6), ^#^*p* < 0.05 versus CM group (*n* = 6). (c) HIF-1*α* in HepG2 cells or HSCs was silenced by sh-RNA. HIF-1*α* and *β*-actin expression levels were determined by immunoblotting. ^∗^*p* < 0.05, sh-control versus sh-HIF-1*α*, *n* = 3. (d) HepG2 or HSCs were treated as in (c), and HIF-1*α* and *β*-actin expression levels were determined by qRT-PCR. ^∗^*p* < 0.05, sh-control versus sh-HIF-1*α*, *n* = 3. All data are representative of at least three independent experiments. (e) Hydrogen peroxide production in HepG2 cells was determined using DCF-DA, and total protein content was used to normalize the data. ^∗^*p* < 0.05 versus St Med group (*n* = 6), ^#^*p* < 0.05 versus CM (*n* = 6).

To investigate the effect of curcumin on HCC-induced angiogenesis, HUVECs were applied to conduct a tube formation assay. As shown in Figures 1(a) and 1(b), conditioned medium from HepG2 + HSCs (CM group) significantly increased tube formation, as compared with conditioned medium from HepG2 cells (St Med group). However, curcumin obviously abolished HSC-enhanced angiogenesis. Intriguingly, NAC, an oxidant scavenger, also abrogated HSC-mediated enhancements of angiogenesis, which indicate that oxidative stress is involved in HSC-enhanced HCC angiogenesis. Moreover, curcumin has a similar oxidant scavenger ability, as it could abrogate ROS production in HepG2 cells induced by HSCs (Figure 1(e)). These data indicate that HSC-induced oxidative stress plays a key role in HCC angiogenesis. And curcumin may inhibit HSC-induced HCC angiogenesis by eliminating ROS production.

Previous study shows that oxidative stress has been largely associated with molecular stabilization of HIF-1*α*. Here, we want to examine whether HIF-1*α* is involved in HCC angiogenesis; we knockdown HIF-1*α* in HepG2 cells using sh-RNA (Figures 1(c) and 1(d)). We found that HSC conditioned medium (CM) could not increase HUVEC tube formation when HIF-1*α* was knockdown in HepG2 cells (Figures 1(a) and 1(b)). Moreover, curcumin or NAC could not influence HUVEC tube formation after HIF-1*α* knockdown in HepG2 cells. In addition, HIF-1*α* knockdown significantly inhibited ROS production in HepG2 cells induced by HSCs. HSC conditioned medium (CM) could not increase ROS production in HepG2 cells when HIF-1*α* was knockdown in HepG2 cells (Figures 1(a) and 1(b)). And curcumin or NAC could not influence ROS production after HIF-1*α* knockdown in HepG2 cells. These data indicate that HSCs induce the proangiogenesis activity of HCC cells. Oxidative stress exhibits a pivotal role in this process. This HSC-induced proangiogenesis in HepG2 cells can be suppressed by curcumin and NAC, and the suppression appears to be dependent on the expression of HIF-1*α*.

### 3.2. Curcumin Abrogates VEGF, IL-6, and SDF-1 Expression in HCC through HIF-1*α*

**Figure 2:**
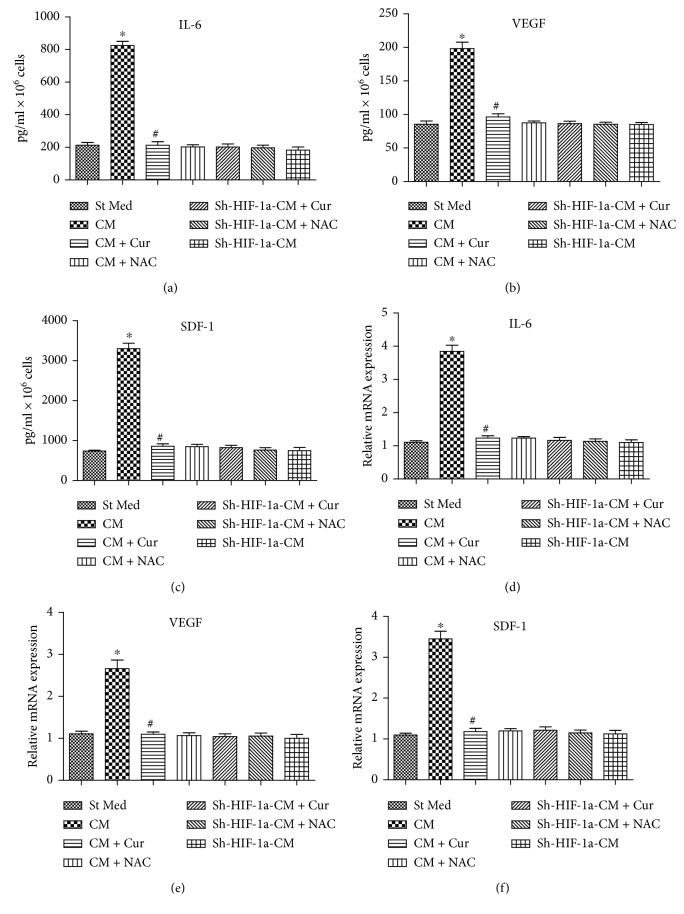
Curcumin decreased VEGF, IL-6, and SDF-1 expression in HCC via inhibiting HIF-1*α*. St Med stands for standard media of PSC cells, CM stands for conditioned media from HepG2 cells, CM + Cur stands for conditioned media from HepG2 cells pretreated with curcumin, CM + NAC stands for conditioned media from HepG2 cells pretreated with NAC, sh-HIF-1*α*-CM + Cur stands for HSC cell knockdown with sh-HIF-1*α* and cultured with conditioned media from HepG2 cells pretreated with curcumin, sh-HIF-1*α*-CM + NAC stands for HSC knockdown with sh-HIF-1*α* and cultured with conditioned media from HepG2 cells pretreated with NAC, and sh-HIF-1*α*-CM stands for HSC cell knockdown with sh-HIF-1*α* and cultured with conditioned media from HepG2 cells. ELISA was assayed to assess IL-6 (a), VEGF (b), and SDF-1 (c) expression in the conditioned medium of the indicated groups. IL-6 (d), VEGF (e), and SDF-1 (f) mRNA expression in HSCs was detected by qRT-PCR, as described in the Materials and Methods. ^∗^*p* < 0.05 versus St Med group (*n* = 6), ^#^*p* < 0.05 versus CM (*n* = 6). All data are representative of at least three independent experiments.

Previous studies suggested that the activated stroma secretes large amounts of IL-6, VEGF, and SDF-1, resulting in a significant enhancement in invasion of the surrounding cancer cells [17–20]. Here, we showed that VEGF, IL-6, and SDF-1 expression levels in HSCs obviously increased after the cells had been cultured in HepG2-derived CM (CM group) (Figure 2). However, curcumin or NAC could abolish the upregulation in VEGF, IL-6, and SDF-1 expression induced by HepG2-derived CM (CM group) (Figure 2), suggesting that curcumin has a similar effect as NAC scavenging oxidative stress to suppress the inflammatory and angiogenic responses in HSCs exposed to HepG2-derived CM.

However, curcumin could not inhibit VEGF, IL-6, and SDF-1 production when HIF-1*α* was knocked down by shRNA in HSCs (Figure 2), suggesting that the inhibition effects of curcumin on VEGF, IL-6, and SDF-1 expression are dependent on HIF-1*α* downregulation (Figure 2).

### 3.3. Curcumin Abolishes HCC EMT and Invasion Induced by HSCs through HIF-1*α*

**Figure 3:**
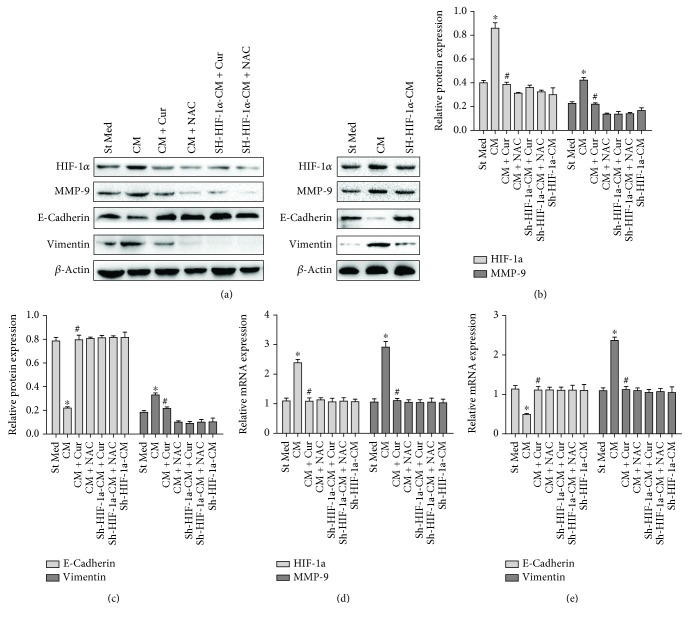
Curcumin abrogated HSC-induced increases in HIF-1*α*, MMP-9 expression and EMT process in HepG2 cells though down-regulating HIF-1*α*. St Med stands for standard media of HepG2 cells, CM stands for conditioned media from HSCs, CM + Cur stands for conditioned media from HSCs with curcumin exposure, CM + NAC stands for conditioned media from HSCs with NAC exposure, sh-HIF-1*α*-CM + Cur stands for sh-HIF-1*α* knockdown HepG2 cells treated with conditioned media from HSCs pretreated with curcumin, sh-HIF-1*α*-CM + NAC stands for sh-HIF-1*α* knockdown HepG2 cells treated with conditioned media from HSCs pretreated with NAC, and sh-HIF-1*α*-CM stands for sh-HIF-1*α* knockdown HepG2 cells treated with conditioned media from HSCs. (A&B&C) HIF-1*α*, MMP-9, E-cadherin, vimentin, and *β*-actin protein expression levels were evaluated by immunoblotting. ^∗^*p* < 0.05 versus St Med group (*n* = 3), ^#^*p* < 0.05 versus CM group (*n* = 3). (d, e) HIF-1*α*, MMP-9, E-cadherin, vimentin, and *β*-actin mRNA expression levels were determined by qRT-PCR. ^∗^*p* < 0.05 versus St Med group (*n* = 3); ^#^*p* < 0.05 versus CM (*n* = 3). All data are representative of at least three independent experiments.

**Figure 4:**
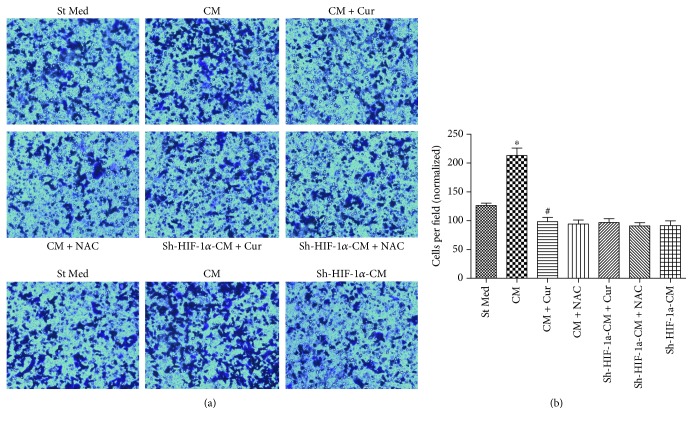
Curcumin suppressed HSC-induced invasion in HepG2 cells through decreasing HIF-1*α*. St Med stands for standard media of HepG2 cells, CM stands for conditioned media from HSCs, CM + Cur stands for conditioned media from HSCs with curcumin exposure, CM + NAC stands for conditioned media from HSCs with NAC exposure, sh-HIF-1*α*-CM + Cur stands for sh-HIF-1*α* knockdown HepG2 cells treated with conditioned media from HSCs pretreated with curcumin, sh-HIF-1*α*-CM + NAC stands for sh-HIF-1*α* knockdown HepG2 cells treated with conditioned media from HSCs pretreated with NAC, and sh-HIF-1*α*-CM stands for sh-HIF-1*α* knockdown HepG2 cells treated with conditioned media from HSCs. The cells were placed in a Matrigel-coated invasion chamber for 20 h. (a, b) We evaluated invasion ability by counting the numbers of migrated cells in ten randomly selected fields under a light microscope at ×100 magnification. ^∗^*p* < 0.05 versus St Med group (*n* = 6), ^#^*p* < 0.05 versus CM (*n* = 6). All data are representative of at least three independent experiments.

Tumor microenvironment exhibits great promotion effects in liver carcinogenesis [11]. Here, we examined whether curcumin could inhibit HSC-induced HCC EMT process and invasion. HepG2 cells were treated with CM from HSCs with or without curcumin or NAC and the expression of associated EMT proteins (e.g., E-cadherin and vimentin) in HepG2 cells were evaluated. Furthermore, a chamber invasion assay was applied to evaluate the invasive ability of the HCC cells. We showed that curcumin or NAC could abrogate the E-cadherin decrease and vimentin increase induced by HSC-derived CM in HepG2 cells (Figure 3). However, we noticed that curcumin or NAC could not influence E-cadherin and vimentin expression when HIF-1*α* was knocked down by shRNA (Figure 3). Similar results were observed in the invasive capacity of HepG2 cells. Curcumin or NAC abolished HSC-derived CM enhanced invasion of HepG2 cell (Figure 4). However, when HIF-1*α* was knocked down in HepG2 cells, curcumin or NAC could not affect HepG2 cell invasiveness (Figure 4). Similar results were found in the expression of MMP-9, an invasion-associated enzyme (Figure 3). These findings indicate that curcumin inhibits HSC-induced HCC invasion, and this inhibition seems to be dependent on oxidative stress and HIF-1*α* expression.

Intriguingly, CM from HSCs could induce HIF-1*α* expression in HepG2 cells, and NAC, a ROS scavenger, significantly reduced HIF-1*α* expression. As CM from HSCs could obviously upregulate ROS production in HepG2 cells, we speculate that ROS may stabilize HIF-1*α* expression to promote HSC-induced HCC invasion.

### 3.4. CTGF Is Responsible for the Observed Effects of HIF-1*α* on HSC Activation and HCC Invasion

**Figure 5:**
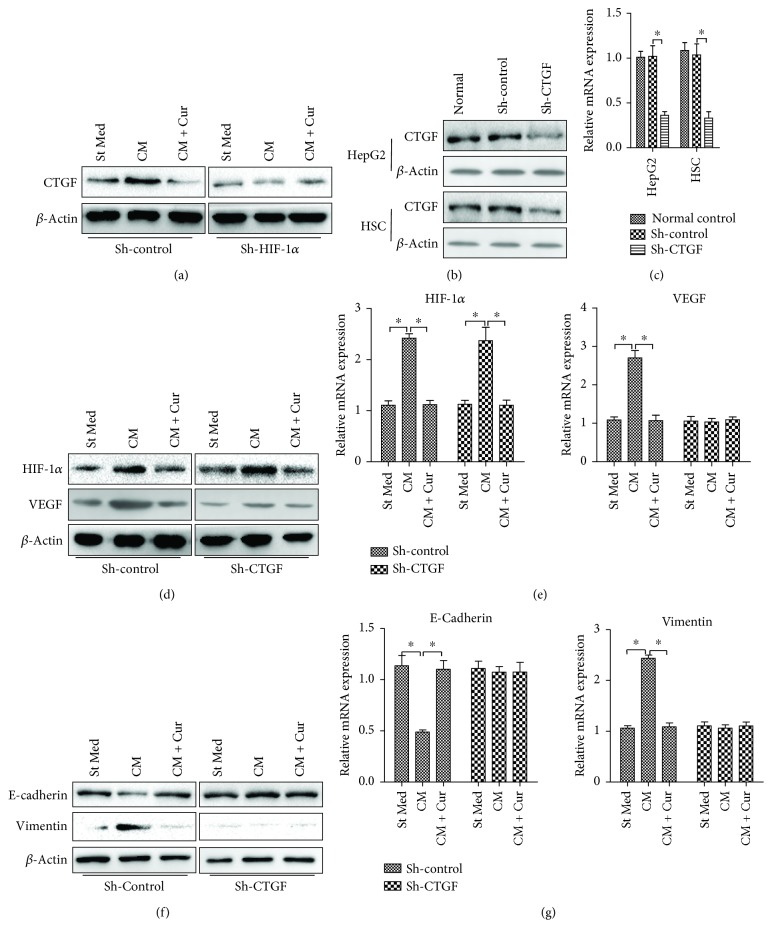
CTGF interference abrogates the observed effects of HIF-1*α* silencing and curcumin on HSC activation and HCC invasion. (a) HepG2 cells were silenced by control shRNA (sh-control) or shRNA targeting HIF-1*α* (sh-HIF-1*α*); CTGF protein levels of HepG2 cells were analyzed by western blot. CTGF interference efficiency in HSCs and HepG2 cells were analyzed by western blot (b) and qRT-PCR (c). (d, e) HSCs transfected with shRNA were cultured with or without curcumin for 12 h and serum starved for an additional 24 h. (d) HIF-1*α* and VEGF protein level of HSCs were analyzed by western blot. (e) HIF-1*α* and VEGF mRNA level of HSCs were analyzed by qRT-PCR. HepG2 cells transfected with CTGF shRNA were incubated with the conditioned media (CM) from HSCs with or without curcumin for 24 h. The cells were lysed, and E-cadherin and vimentin expression levels were analyzed by western blot (f) and qRT-PCR (g). ^∗^*p* < 0.05. All data are representative of at least three independent experiments.

As shown in Figure 5(a), CM derived from HSCs could increase CTGF expression in HepG2 cells, which could be inhibited by curcumin. When HIF-1*α* was knocked down in HepG2 cells, CTGF expression decreased significantly. And CM derived from HSCs could not affect CTGF expression after HIF-1*α* interference. In tumor cells, CTGF has been reported to regulate growth, migration, invasion, and angiogenesis [21]. We investigated whether CTGF is responsible for the observed effects of curcumin and HIF-1*α* on HSC activation and HCC invasion. CTGF shRNA was used to target CTGF expression in both HSCs and HepG2 cells (Figures 5(b) and 5(c)). And then the HIF-1*α* and VEGF expression in HSCs and the E-cadherin and vimentin expression in HepG2 cells were tested. CTGF shRNA significantly suppressed VEGF expression in HSCs (Figure 5(d)). However, HIF-1*α* expression was not affected by CTGF shRNA (Figures 5(d) and 5(e)). Moreover, CTGF knockdown in HepG2 cells increased E-cadherin expression and decreased vimentin expression in HepG2 cells cultured with CM from HSCs (Figures 5(f) and 5(g)). Furthermore, when CTGF was knocked down in HepG2 cells, curcumin could not affect HSC activation or HepG2 cell invasiveness (Figure 5). Since CTGF shRNA could not influence HIF-1*α* expression in HSCs and HIF-1*α* knockdown could downregulate CTGF expression, these data indicate that CTGF is a downstream gene of HIF-1*α* and is responsible for the observed effects of curcumin and HIF-1*α* on HSC activation and HCC invasion.

### 3.5. Curcumin Induces Nrf2 and GSH Expression in HCC Protection

**Figure 6:**
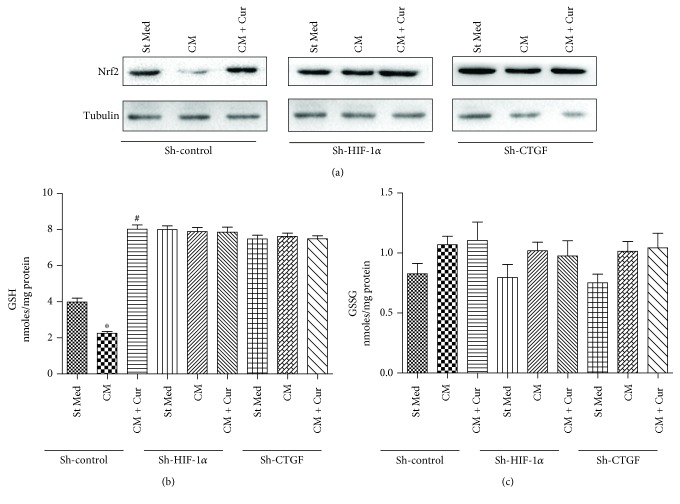
Nrf2 and GSH participate in curcumin-induced HCC protection. (a) HepG2 cells were silenced by control shRNA (sh-control), shRNA targeting HIF-1*α* (sh-HIF-1*α*), or shRNA targeting CTGF (sh-CTGF); nuclear Nrf2 protein levels of HepG2 cells were analyzed by western blot. (b) Glutathione (GSH) and glutathione disulfide (GSSG) levels were evaluated in HepG2 cells. ^∗^*p* < 0.05 versus St Med group (*n* = 3), ^#^*p* < 0.05 versus CM (*n* = 3). All data are representative of at least three independent experiments.

To elucidate possible mechanisms of HCC protection by curcumin, we tested nuclear Nrf2 and total GSH and GSSG expression in HepG2 cells. As shown in Figure 6, curcumin induced significant Nrf2 and GSH expression in HepG2 cells without affect GSSG expression. However, when HIF-1*α* or CTGF was knocked down in HepG2 cells, curcumin could not influence Nrf2 or GSH expression. These data indicate that curcumin may induce ARE by upregulating Nrf2 and GSH expression in HCC protection. This effect is dependent on HIF-1*α* and CTGF expression.

## 4. Discussion

As is well known, HCC stroma and peritumoural tissue were infiltrated with activated HSCs, and HSCs are located at tumor sinusoids, tumor capsule, and fibrous septae [7, 22, 23]. Moreover, activated HSCs have also been found in the periphery of dysplastic nodules within the liver [24]. In response to liver injury, quiescent HSCs activated into matrix-secreting myofibroblasts and are the major producer of ECM proteins in the process of liver fibrogenesis [25–27]. As master regulators of fibrosis, HSC may hence directly affect HCC formation through effects on the tumor stroma. In addition, the interaction between tumors and cancer-associated fibroblasts is well established in other systems that complex intercellular signaling networks is involved in this process, contributing to cancer initiation, growth, and progression [26, 28–31]. In our study, we added evidence that HSCs promoted HCC oxidative stress, angiogenesis, invasion, and EMT process. ROS and HIF-1*α* exhibit very important function in mediating the HSC and HCC cell interplay. CTGF is responsible for HIF-1*α* effects on HSC activation and HCC invasion.

VEGF, SDF-1, and CTGF, which are associated with angiogenesis and chemoattraction of cancer and endothelial cells, and IL-6, which is associated with the proinflammatory response, have already been proven to be a downstream gene of HIF-1 [32, 33]. Our recent studies have shown that exogenous SDF-1 could increase CXCR4-positive pancreatic cancer invasion and EMT [34], and activated pancreatic cancer stellate cells could secrete SDF-1 and IL-6 to induce EMT in pancreatic cancer [18]. This study revealed that coculture of HepG2 and HSCs elicited much more VEGF, SDF-1, and IL-6 secretion in HSCs, suggesting that HCC cells surrounded by HSCs may more likely metastasize to other sites than other cells. Therefore, activated HSCs are active players in attracting hepatocarcinoma cells to different locations. Active factors in this chemoattraction include CTGF, SDF-1, VEGF, and IL-6, confirming their pleiotropic role in hepatocarcinoma progression. Hence, the surrounding stroma might play a role in attracting metastatic hepatocarcinoma cells from the primary lesions, thereby facilitating satellite metastases.

Angiogenesis is closely related to HCC initiation, progression, and metastasis [35], as sorafenib could efficiently target these processes [36, 37]. Multiple proangiogenic factors stimulate new vessel formation to sustain the rapid growth pattern of malignant hepatocytes which in turn facilitates tumor progression and metastasis [38]. However, the molecular mechanisms underlying angiogenesis remain poorly understood [39]. In our study, we revealed that HSCs promoted tube formation and VEGF expression via upregulating HIF-1*α* expression, suggesting that HIF-1*α* is a potential target for HCC therapy. Furthermore, curcumin inhibited tube formation and VEGF expression, and knockdown of HIF-1*α* abrogated these effects, suggesting that curcumin has prominent therapeutic effects on HCC through targeting HIF-1*α*. In addition, CTGF is a downstream gene of HIF-1*α* and is responsible for the observed effects of curcumin and HIF-1*α* on HSC activation and HCC invasion.

Curcumin and NAC eliminated ROS production in HCC cocultured with HSCs, and also suppressed HCC progression, suggesting that ROS plays a key role in curcumin inhibitory effect on HCC. ROS is significantly associated with tumor aggression via several pathways. They can regulate the activity of transcription factors through inducing DNA damage and genome instability and can also affect gene expression. Also, ROS production is associated with EMT process in several tumors [18, 40, 41]. Here, we showed that curcumin induced Nrf2 and GSH expression without affecting GSSG expression. Nrf2 and GSH are well known to have ability to induce antioxidant response element (ARE). Thus, curcumin may induce ARE by upregulating Nrf2 and GSH expression. However, curcumin could not influence Nrf2 and GSH expression when HIF-1*α* or CTGF was knocked down, as curcumin could inhibit HIF-1*α* expression and CTGF is a downstream gene of HIF-1*α*. These data indicate that curcumin may induce ROS scavenging by upregulating Nrf2 and GSH, thus inhibiting HIF-1*α* stabilization to suppress CTGF expression to exhibit its protection on HCC.

It has been shown that curcumin has protective potential in multiple human carcinomas including prostate, head and neck, melanoma, breast, colon, and pancreatic cancers [6], such as inhibiting cancer growth, metastasis, and increasing chemopreventive effect of other anticancer medicines [16, 42, 43]. Epidemiological studies revealed that the low incidence of colon cancer in India is due to the chemopreventive and antioxidant properties of curcumin [44]. The underlying mechanisms of its anticancer effects are comprehensive and diverse. Our data revealed that curcumin suppressed IL-6 and SDF-1 expression and ROS production and inhibited HCC invasion. Moreover, our results suggest that curcumin inhibits VEGF expression to reduce HCC angiogenesis. However, VEGF, IL-6 expression or ROS production could not be inhibited by curcumin when HIF-1*α* was knocked down in HSCs, which suggest that HIF-1*α* is a vial factor in curcumin-mediated inhibition of HCC progression. Furthermore, CTGF is a downstream gene of HIF-1*α* and is responsible for the observed effects of curcumin and HIF-1*α* on HSC activation and HCC invasion.

## Data Availability

The data used to support the findings of this study are included within the article.
